# Bio-Inspired Functional Surfaces Based on Laser-Induced Periodic Surface Structures

**DOI:** 10.3390/ma9060476

**Published:** 2016-06-15

**Authors:** Frank A. Müller, Clemens Kunz, Stephan Gräf

**Affiliations:** Otto Schott Institute of Materials Research (OSIM), Löbdergraben 32, Jena 07743, Germany; clemens.kunz@uni-jena.de (C.K.); stephan.graef@uni-jena.de (S.G.)

**Keywords:** bio-inspired materials, functional surfaces, laser-induced periodic surface structures, ultra-short laser pulses, wettability, superhydrophobicity, optical properties, antireflective surfaces, structural colors, tribology

## Abstract

Nature developed numerous solutions to solve various technical problems related to material surfaces by combining the physico-chemical properties of a material with periodically aligned micro/nanostructures in a sophisticated manner. The utilization of ultra-short pulsed lasers allows mimicking numerous of these features by generating laser-induced periodic surface structures (LIPSS). In this review paper, we describe the physical background of LIPSS generation as well as the physical principles of surface related phenomena like wettability, reflectivity, and friction. Then we introduce several biological examples including e.g., lotus leafs, springtails, dessert beetles, moth eyes, butterfly wings, weevils, sharks, pangolins, and snakes to illustrate how nature solves technical problems, and we give a comprehensive overview of recent achievements related to the utilization of LIPSS to generate superhydrophobic, anti-reflective, colored, and drag resistant surfaces. Finally, we conclude with some future developments and perspectives related to forthcoming applications of LIPSS-based surfaces.

## 1. Introduction

In the course of evolution, animals and plants have developed numerous skills and structures to ensure their survival. For this purpose, nature continuously optimized the properties of materials via natural selection. Sophisticated hierarchical structures (e.g., bone, beaks, nacre, wood) and fibers (e.g., spider silk, polar bear hair, mussel byssus) were developed to generate e.g., functional light-weight structures with outstanding mechanical properties. Beyond that, numerous functional surfaces were developed to solve very different specific problems like e.g., wettability (e.g., lotus leaf), friction and wear (e.g., pangolin skin), antifouling (e.g., shark skin), reflectivity (e.g., butterfly wings), and adhesion (e.g., gecko feet). Consequently, bio-inspired principles have attracted increasing attention in materials science and engineering. Related activities aim on learning from nature how to develop technical systems whose function simulates natural systems and how to prepare artificial materials and surfaces with so far unrivalled properties. For this purpose, numerous different physical, chemical and mechanical methods are available to synthesis functional surfaces. Over the last decades, the use of lasers in materials processing has attracted continuously increasing attention due to the high cost efficiency and excellent quality of processed products. In industrial production, lasers are successfully used as drilling and cutting tools and to weld materials together. They are also utilized in additive manufacturing technologies (e.g., stereolithography, selective laser sintering) that are on their way from research to industry. Beyond, they may initiate gas phase condensation processes to synthesize nanoparticles (e.g., laser vaporization) and coatings (e.g., pulsed laser deposition). In recent years, ultra-short laser beams that are pulsed in the fs to ps range were used to prepare laser-induced periodic surface structures (LIPSS). These structures attracted particular interest in research and development because of their great potential to create manifold functional surfaces for various technical applications. Here, we will give a comprehensive overview on the generation of LIPSS and on their exploitation to mimic functional biological surfaces.

## 2. Laser-Induced Periodic Surface Structures

In the year 1965, Birnbaum was the first to obtain laser-induced periodic surface structures (LIPSS) by utilizing pulsed ruby lasers to irradiate semiconductors [1]. Since then extensive research was carried out to investigate the formation mechanism of these structures. In the meanwhile, LIPSS have been identified as a universal phenomenon that can be observed on all classes of materials when irradiated near their ablation threshold [2]. LIPSS fabrication has mainly been demonstrated on flat substrates and more recently also on the curved surface of carbon fibers whose curvature is in the order of the incident laser wavelength [3]. Generally, it was shown, that LIPSS evolution and properties are influenced by a great variety of influencing parameters including e.g., the wavelength *λ* and the fluence *F* of the laser radiation, the optical properties of the material and the number *N* of laser pulses hitting the materials surface. With respect to the wide range of these determining factors, the key-parameter is related to the laser beam polarization. This strong influence is particularly expressed by the degree of order/disorder as well as the alignment of the emerging pattern and was investigated in detail by numerous authors by using linear, elliptical and circular polarization [4,5,6,7,8,9].

**Figure 1:**
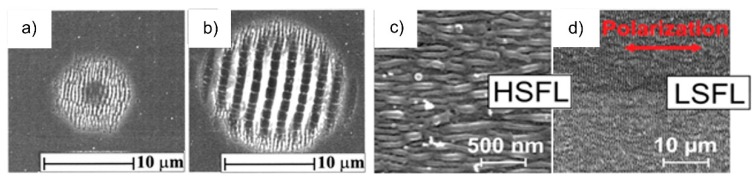
SEM micrographs of laser-induced periodic surface structures: (**a**) HSFL and (**b**) LSFL on InP and (**c**) HSFL and (**d**) LSFL on titanium. (**a**,**b**) Reprinted from Borowiec and Haugen [19] and (**c**,**d**) from Bonse *et al*. [2], with the permission of AIP Publishing.

During the illumination of the surface of materials with linearly polarized radiation, the emerging structures are classified with regard to their period *Λ* into low-spatial frequency LIPSS (LSFL) and high-spatial frequency LIPSS (HSFL) (Figure 1). LSFL are predominantly characterized by an orientation perpendicular to the electric field (E-field) vector. Exceptions are related to some dielectric materials (e.g., fused silica, BaF_2_) and certain polymers, where LSFL are aligned parallel to the laser beam polarization [2,10,11,12]. Upon irradiation of strong absorbing materials such as metals and semiconductors by fs-laser radiation, usually LSFL are observed with *Λ* close to the incident laser wavelength *λ* [2,13,14,15,16,17,18]. Similar behavior was reported for certain polymers irradiated with higher harmonics of a Ti:sapphire fs-laser due to the occurrence of absorption bands in the corresponding spectral range [12]. Spatially modulated intensity pattern resulting from interference effects of the incident laser radiation with surface electromagnetic waves are mainly used to explain the phenomenon of LSFL formation [4].

In this context, the excitation of surface plasmon polaritons (SPP) as well as the influence of surface roughness and isolated defects have to be considered [20,21,22]. The different behavior of metals, semiconductors and insulators is determined by their electronic structure and the incident laser wavelength *λ*. In the case of dielectrics such as glasses, ceramics and polymers, the absorption of the high intensive fs-laser radiation leads to a metal-like behavior that results from the highly excited state of the material. In this case, LSFL emerge with *Λ* either close to *λ* or close to *λ*/*n* with *n* being the corresponding refractive index of the material. A comprehensive overview of HSFL reported on inorganic solids is given by Bonse *et al*. [2]. They are usually observed for the irradiation with fs-laser pulses, mainly for below band-gap excitation of transparent materials [2,23], but also on metals [2]. HSFL are characterized by a period much smaller than *λ* and an orientation that can be parallel or perpendicular to the linear beam polarization in dependence on the type of material. Their origin, however, still remains unclear and hence numerous investigations are still under research. Possible explanations include self-organization [24], second-harmonic generation [19] and chemical surface alterations (e.g., oxidation) [25]. It has to be noted, that contrary to linearly polarized radiation, circular polarization at normal incidence leads to the formation of random structures consisting of nanodots and ripples with many intersections and bifurcations [5,6,7]. Moreover, some authors showed that elliptical polarization leads to a LIPSS pattern with an orientation perpendicular (e.g., for CaF_2_) [6,7] or parallel (for certain polymers) [26] to the long axis of the polarization ellipse.

## 3. Wettability

### 3.1. Theoretical Aspects

**Figure 2:**
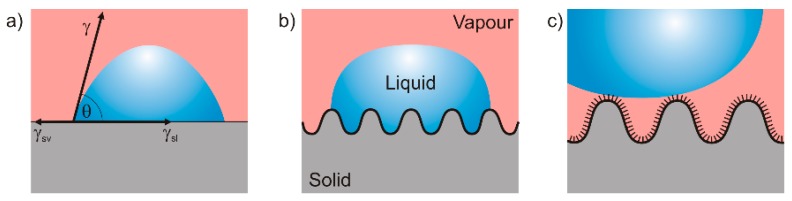
Wetting states (**a**) Young; (**b**) Wenzel; and (**c**) Cassie-Baxter.

The wetting behavior of a solid surface depends on the surface energy *γ* of the liquid, the interfacial energy *γ_sv_* between solid and vapor, and the interfacial energy *γ_sl_* between solid and liquid (Figure 2a). The intrinsic contact angle *θ* of a smooth and inert surface can then be described by Young’s equation [27]:

(1) $$\cos\theta =\frac{\gamma_{sv}-\gamma_{sl}}{\gamma }$$

By convention, surfaces with a contact angle *θ* < 90° are referred to as wetting, those with *θ* > 90° as non-wetting. In the case of the liquid water, wetting and non-wetting surfaces are referred to as hydrophilic and hydrophobic, respectively. If *θ* exceeds 150° surfaces are designated superhydrophobic. However, on rough surfaces the measured contact angle often differs from the intrinsic one. This was first described by Wenzel in 1936 [28], who implemented a roughness factor *r* > 1, which represents the ratio of the true surface area of the solid to its horizontal projection (Figure 2b). With this factor, the roughness dependent Wenzel contact angle *θ^W^* can be calculated as follows:

(2) $$\cos\theta^{W}=r\times \cos\theta$$

Thus, Wenzel’s geometrical model allows explaining why the contact angle of hydrophilic surfaces decreases with increasing roughness whereas the contact angle of hydrophobic surfaces increases. However, Wenzel’s model can hardly explain water contact angles exceeding 150°. This behavior requires an additional model that was given by Cassie and Baxter in 1944 [29]. Here, the surface tension *σ* dominates the system and it is energetically favorable for the liquid to form a droplet that rests on the tips of the surface while air fills the space between solid and liquid (Figure 2c). Consequently, only a small fraction of the surface *φ_s_* is in contact to the liquid. The Cassie-Baxter contact angle *θ^CB^* can then be calculated according to:

(3) $$\cos\theta^{CB}=\phi_{S}\times \cos\theta +\phi_{S}-1$$

According to Cassie-Baxter´s wetting state, water that is in contact to a superhydrophobic surface forms droplets that can easily unroll a surface at very low angles of inclination. On their way they wet contaminating particles present on the surface because thereby the surface energy of the droplet is reduced and energy is gained by adsorption. Thus, the droplet picks up the particles and removes them from the surface. This self-cleaning ability of a superhydrophobic surface is also called “Lotus-Effect” [30] because these observations have already been documented in early Sanskrit writings describing the purity of lotus leafs.

### 3.2. Examples in Nature

**Figure 3:**
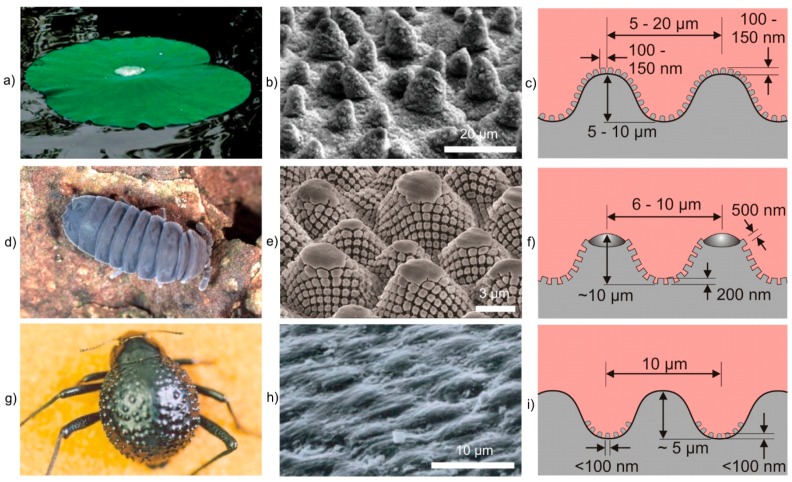
Examples of superhydrophobic surfaces in nature: (**a**–**c**) leaves of the lotus lower *Nelumbo nucifera*; (**d**–**f**) *Tetrodontophora bielanensis* springtail cuticle and (**g**–**i**) Namib dessert beetle *Stenocara gracilipes*; (**a**,**b**) Hensel *et al*. [33]—Published by The Royal Society of Chemistry; (**d**,**e**) Adapted by permission from Macmillan Publishers Ltd.: NPG Asia Materials, Hensel *et al*. [31], copyright 2013; (**g**,**h**) Adapted by permission from Macmillan Publishers Ltd.: Nature, Parker and Lawrence [32], copyright 2001.

Numerous plants (e.g., lotus leaf) [30] (Figure 3a–c) and living creatures (e.g., springtail cuticle) [31] (Figure 3d–f) have developed superhydrophobic surfaces by combining hydrophobic materials (e.g., long-chain fatty acids, waxes) with surface profiles that are hierarchically structured by combining a waviness on the microscale with a roughness on the nanoscale. The investment in generating such complex structures mainly aims on keeping the surface clean and dry, which is a requirement to protect the surface from the colonization of germs and microorganisms and to prevent the clogging of breathing pores. Beyond, there are examples for surfaces that combine hydrophilic and hydrophobic regions (e.g., Namib desert beetle) [32] (Figure 3g–i). Here, the hydrophilic peaks of bumps facilitate the condensation of water from fog, while the superhydrophobic troughs provide a pathway for the water droplets to roll towards the mouthparts of the beetle.

### 3.3. Superhydrophobic LIPSS

Wettability control and superhydrophobicity of material surfaces are probably the most studied examples of bioinspired materials research in the past. Consequently, a broad spectrum of technical applications is given including products like self-cleaning [34], anti-fingerprint [35], anti-corrosion coatings [36], water-repellent textiles [37], the prevention of bio-fouling [38] and fog harvesting [39]. It has been demonstrated, that the wetting behavior is not only a matter of the surface chemistry alone. More importantly, it was shown on example of the hierarchical morphology of the lotus leaf (Figure 3a–c), that the surface morphology directly affects the wetting features [40,41]. In addition to a contact angle *θ* exceeding 150° a superhydrophobic surface is characterized by a sliding angle (SA) below 5° [28,29]. The production of these surfaces has been demonstrated by means of numerous different techniques like photolithography [42], laser interference lithography [43], and chemical etching [44]. Readers with a deeper interest are referred to comprehensive reviews given by Roach *et al*. [45] and Wang *et al*. [46]. However, main disadvantages of these techniques are related to the requirement for multiple steps and additional materials as well as to their intensive time and cost factor [47].

**Figure 4:**
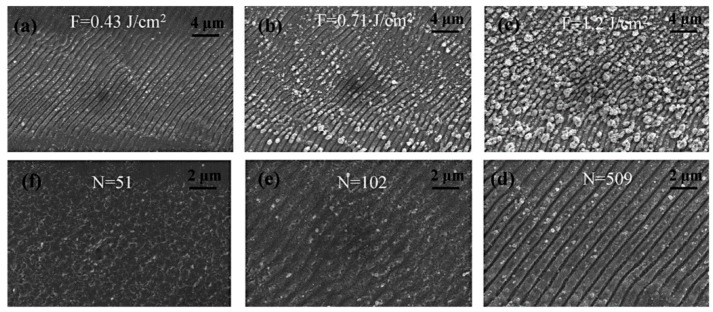
LIPSS fabricated on copper surfaces with (**a**–**c**) different laser fluences *F* at fixed pulse number *N* = 509 and (**d**–**f**) with different number of pulses *N* at *F* = 0.43 J/cm^2^. Reprinted from Long *et al*. [49], Copyright 2014, with permission from Elsevier.

Generally, the fabrication of superhydrophobic surfaces has to be distinguished into the two following strategies: The first approach focuses on the increase of the surface roughness of a raw material with a low surface energy. The second concept involves the increase of the surface roughness to obtain a hydrophilic intermediate state in combination with a post-chemical surface functionalization to achieve the finale hydrophobic wettability behavior [48]. The latter method has been studied intensively, particularly concerning the transfer of hydrophilic to hydrophobic metal surfaces [49,50,51]. Regarding the fabrication of superhydrophobic surfaces, the irradiation with ultrashort laser pulses offers a flexible, robust and direct technique. Beyond, chloroalkysilane and fluoroalkysilane coatings are frequently used to increase *θ* to superhydrophobic states with decreasing surface energy [52]. It has been shown, that a densely packed CF_3_ functionalized flat surface can reach a theoretical contact angle of *θ* = 120°. Increasing the surface roughness with LIPSS, *θ* exceeding 120° can be realized after silanization [53,54]. Long *et al*. [49] investigated the influence of the laser fluence F and the pulse number *N* on the resulting morphology of LIPSS fabricated on copper surfaces using a ps-laser with *λ* = 1064 nm (Figure 4). It becomes evident, that for all investigated parameters, the structural period *Λ* was in the same range between 700 nm and 800 nm. Varying *F* between 0.43 J/cm^2^ and 1.2 J/cm^2^ at a fixed pulse number *N* = 509, an increasing size and density of nanoparticles loaded on the LIPSS pattern was demonstrated (Figure 4a–c). In addition, an increasing *N* at *F* = 0.43 J/cm^2^ led to an increasing depth, *i.e.*, to a more pronounced grating structure (Figure 4d–f). However, at this low fluence the corresponding surfaces exhibit only a small number of nanoparticles. The subsequent reduction of the surface energy based on the functionalization with triethoxyoctylsilane resulted in the increase of *θ* up to 154° with increasing *F* used during LIPSS fabrication. The hydrophobicity was attributed to an increased surface roughness caused by the nanoparticles, whereas the smallest *θ* was achieved using the lowest laser fluence.

**Figure 5:**
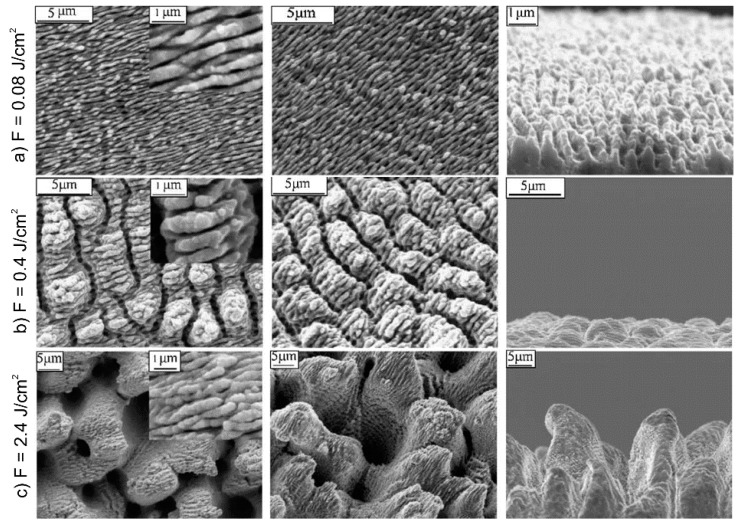
SEM micrographs of top, side (30°) and profile view of stainless steel surfaces at various laser fluence: (**a**) *F* = 0.08 J/cm^2^; (**b**) *F* = 0.4 J/cm^2^; and (**c**) *F* = 2.4 J/cm^2^. Reprinted from Wu *et al*. [51], Copyright 2009, with permission from Elsevier.

The influence of high laser fluences (*i.e.*, far above the ablation threshold) on the fs-laser irradiation of metallic surfaces was demonstrated by several authors [50,51]. Starting from conventional LIPSS fabricated with *F* = 0.08 J/cm^2^ on stainless steel (Figure 5a), Wu *et al*. observed an increasing depth and the occurrence of a new groove structure perpendicular to the initial LIPSS orientation by increasing *F* up to 0.4 J/cm^2^ (Figure 5b) [51]. A further increase of *F* resulted in microscale “cone-like” structures with LIPSS on the top (Figure 5c) that are phenomenologically characterized by a very rough surface and a high degree of disorder.

**Figure 6:**
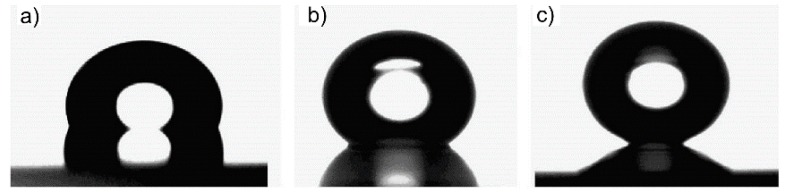
Photographs of water droplets on (**a**) flat; (**b**) LIPSS and (**c**) double-scale structure stainless steel surfaces after silanization. Reprinted from Wu *et al*. [51], Copyright 2009, with permission from Elsevier.

After laser treatment, the surfaces were immediately silanized by Trichloro(1,1,2,2-perfluorooctyl)silane. Compared to a non-structured surface with *θ* = 105° ± 3° (Figure 6a), LIPSS induced by irradiation at low *F* exhibit *θ* = 150.3° (Figure 6b). The maximum contact angle *θ* = 166.3° together with a small SA of 4.2° was achieved at high *F* (Figure 6c). Consequently, the surfaces show extremely superhydrophobic properties, which are caused by air pockets due to the rough surface and subsequent silanization [51].

**Figure 7:**
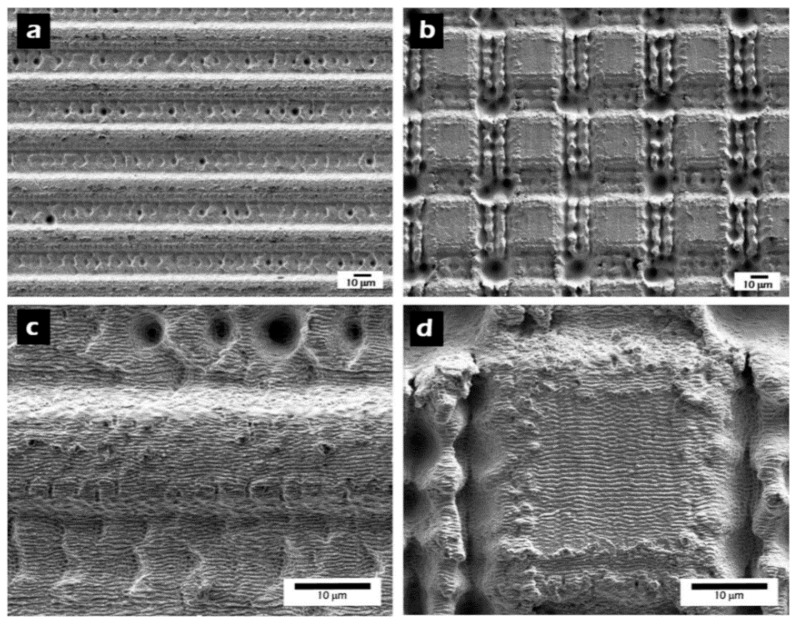
SEM micrographs of hierarchical structures fabricated on stainless steel: (**a**) trench micro pattern with a pitch distance of 30 µm; and (**b**) matrix micro pattern with a pitch distance of 50 µm both covered by LIPSS. Detailed area scanned on: (**c**) trench micro pattern; (**d**) matrix micro pattern. Reprinted from Martínez-Calderon *et al*. [47], Copyright 2015, with permission from Elsevier.

Hierarchical structures consisting of LIPSS on the top of well-ordered micro-structured stainless steel surfaces were demonstrated by Martínez-Calderon *et al*. who investigated the influence of these “hybrid” structures on *θ* [47]. For this purpose, trench as well as matrix micro-pattern with a depth of around 10 µm and line widths of about 20 µm were realized by using fs-laser radiation (Figure 7). The superimposed LIPSS are characterized by *Λ* = 580 nm and a height of about 250 nm.

**Figure 8:**
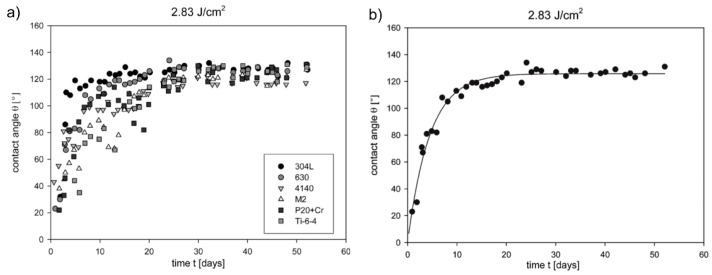
Evaluation of the time-dependency of *θ* of surfaces irradiated with *F* = 2.83 J/cm^2^: (**a**) *θ* of different substrate materials and (**b**) exponential growth regression on *θ*-evaluation in the case of stainless steel. Adapted with permission from Kietzig *et al*. [40], Copyright 2009 American Chemical Society.

Based on the contact angle *θ* = 75° of the untreated flat stainless steel surface, *θ* was enhanced to a maximum value of 144° by structuring the surfaces with trenches and matrix micro patterns without LIPSS. Covering these microstructures with LIPSS led to an increase of *θ* up to 156°, *i.e.*, to a highly hydrophobic surface. Experiments concerning the influence of the pitch distance on *θ* revealed a decrease of *θ* with increasing distance. As a result, a maximal *θ* can be realized by LIPSS containing a dual scale structure allowing to reach the Cassie-Baxter state [47]. It has to be noted, that based on these hierarchical structures, hydrophobic surfaces can be obtained from an initially hydrophilic surface without additional functionalization (e.g., coatings, silanization or additional treatments). Beyond the morphological point of view, the investigations of Kietzig *et al*. revealed a change of *θ* of LIPSS-based surfaces of different materials over time [40,55] (Figure 8). This aging effect leads to a change of the wettability from hydrophilic immediately after laser structuring to hydrophobic with increasing time. Beyond, the time required for this transfer increases with increasing fluence [40].

Based on XPS measurements of the untreated surfaces as well as of surfaces immediately after laser treatment and after 52 days, the time-dependency of *θ* was attributed to the enrichment of carbon at the structured surfaces resulting from the decomposition of carbon dioxide by the intensive fs-laser pulses. However, the amount of carbon from the initial decomposition is not sufficient to cover the whole surface and to protect the underlying iron oxide. This polar iron oxide and the surfaces roughness according to the Wenzel theory support the wetting behavior towards hydrophilicity. Over time, the decomposition slowly proceeds and the content of non-polar carbon increases. With increasing carbon content, the wettability of the structure with dual scale roughness transforms to hydrophobic. These explanations were confirmed by the storage of irradiated samples in different media. As a main result, a carbon dioxide atmosphere led to a faster change of *θ* when compared to normal air atmosphere. On the other hand, *θ* remained unaffected when the samples were stored under water [40]. Although this theory is the most cited in literature, the “aging effect” is discussed controversially. Some other groups like Bizi-bandoki *et al*. [41] propose that for aluminum the time-dependent change to hydrophobic behavior is related to the laser-induced formation of new non-polar carbon groups like –CH_3_ and graphitic carbon. In the case of stainless steel, water molecules were detected instead of functional groups, which disappeared after laser processing and made the structured surfaces less polar, when compared to the initial surfaces. In conclusion, Bizi-bandoki *et al*. predict the topography effect (roughness) to be the dominating factor immediately after laser processing. Over time, the surface chemistry becomes predominant and leads to hydrophobic stainless steel surfaces [41].

As an indirect LIPSS-based technique for the production of superhydrophobic surfaces, the replica method provides the possibility to transfer the morphology of fs-laser structured metal surfaces on polymeric materials. In this context, Liu *et al*. [56] investigated the wettability of a structured master material (stainless steel), the resulting replica (polydimethylsiloxane) and of replicas sputtered with gold. They showed, that a superhydrophobic behavior with self-cleaning effects (*θ* = 164.5°, SA = 8.44°) could be realized on the PDMS replica. Based on a remarkable decrease of *θ* related to the same replicas sputtered with a 300 nm thick gold layer (*i.e.*, the sub-micro/nano-structures are eliminated), they confirmed the roughness and the hierarchical structure, respectively, as the reason for the superhydrophobicity.

## 4. Reflectivity

### 4.1. Theoretical Aspects

The reflectivity *R* of a perfectly flat material’s surface describes the fraction of the incident intensity of light that is reflected at an interface between a surrounding medium and a material. *R* depends on the wavelength *λ* and on the angle of the incident light, on its polarization, and on the optical constants *n_m_* (refractive index) and *κ_m_* (absorption index) of the material. In the case of a normal incidence and a non-absorbing medium (e.g., air with *n*_0_ = 1), *R* is given by:

(4) $$R=\frac{{\left(n_{m}-1\right)}^{2}+n_{m}^{2}\kappa_{m}^{2}}{{\left(n_{m}+1\right)}^{2}+n_{m}^{2}\kappa_{m}^{2}}$$

Anti-reflective coatings aim on minimizing *R* by maximizing either the part of the absorption *A* or the transmission *T*:

(5) R=1−A−T

The simplest procedure to reduce the reflectivity *R* of e.g., optical crown glass (*n_m_* = 1.52 and *κ_m_* ≈ 0 at *λ* = 500 nm; *R* = 4.3%) is to coat it with a thin layer (*λ*/4) of a material whose refraction index *n_c_* lies between *n*_0_ and *n_m_*. The optimum value of *n_c_* (=1.23 for the present example) can be calculated by using [57]:

(6) $$n_{c}=\sqrt{n_{0}\times n_{m}}$$

**Figure 9:**
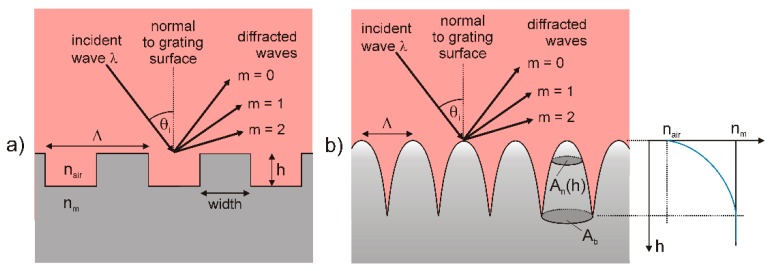
Schematic illustration of a plane wave incident on a periodic surface grating with (**a**) rectangular groove profile and (**b**) gradient profile.

An alternative method to reduce the reflection of light is given by textured surfaces, where gratings consisting of 3D pyramids or 2D grooves interact with the incident light [58]. Equation (7) describes the condition for a constructive interference from periodic elements on a surface [59]

(7) $$m\times \lambda =\Lambda \times \left(\sin\theta_{i}\times \cos\phi +\sin\theta_{m}\right)$$

with the incident angle *θ_i_*, the diffracted angle *θ_m_* for order m, the orientation *φ* of the ripple pattern relative to the plane of incidence, the wavelength *λ* and the groove spacing *Λ* (Figure 9a). These parameters control the possible presence and direction of various orders. However, the efficiency of the grating is determined by the wavelength and polarization of incident light, the height *h* and shape of the grooves, and the optical properties of the grating surface [60].

Depending on the *λ*/*Λ* ratio, a reduction of the reflection can be described theoretically [58]. If the wavelength exceeds the structural size of the grating (*λ*/*Λ* > 1), the grating behaves like a gradient index film with reduced reflection [61,62]. In this case, the reflection can be calculated by using effective medium approximations. If the wavelength is smaller than the structure size of the grating (*λ*/*Λ* < 1), rays should be reflected many times before they revert [63]. In this case, the reduction of R can be explained by a geometric optics approximation, and *R* can be calculated by ray tracing [64]. In the case of similar sizes of wavelength and structure (*λ*/*Λ* ≈ 1), no valid approximations exist to calculate the reduction of *R*. The reflection can only be calculated by solving Maxwell’s equations numerically [58]. However, it was shown that the reflection decreases when the height of the structure is increased and the dielectric mismatch between the grating and the incident medium is reduced [65,66].

A special case occurs if the grating has a nipple-like gradient profile with a gradual change of index (Figure 9b). Then, the regarding reflectance is a result of an infinite series of reflections at each incremental change in index [67]. Since each reflection comes from a different depth of the surface, each will have a different phase. If the transition takes place over an optical distance of *λ*/2, all phases are present, there will be destructive interference and *R* will fall to zero [67]. The effective refractive index *n_eff_* can be calculated with [68]

(8) $$n_{eff}\left(h\right)=\frac{n_{m}\times A_{n}\left(h\right)}{A_{b}}+\frac{A_{b}-A_{n}\left(h\right)}{A_{b}}$$

where *A_n_*(*h*) is the area of the nipples as a function of their height, and *A_b_* is the base area of a nipple. Beyond, the interaction of visible light with a textured surface can be used to optimize the reflection of light of selected wavelengths and to produce structural colors. It is known, that a number of structures can create structural colors, by mechanisms including interference, diffraction gratings, scattering, and photonic crystals [69]. The resulting colors originate from the selective reflectance and absorption of incident light, and they may be affected by the angle of light incidence. Diffraction gratings divide white light into spectra. Rays scattered from different points on the grating interfere either constructively or destructively [70]. Depending on the geometry of the grating, this may lead to characteristic structural colors of high brightness. Photonic crystals are another important source of structural coloration. The sub-wavelength lattices of these periodic 1D, 2D, or 3D structures can control the propagation of light in a manner atomic crystals control electrons [71]. Photonic crystals with a 2D and 3D structure may be regarded as a special case of composite, built from two materials with refractive indices *n_m_*_1_ and *n_m_*_2_, characterized by a refractive index invariant under the spatial translations of a crystalline lattice [69]. Thus, photonic crystals can be defined as a medium with a refractive index that varies in space periodically [72].

### 4.2. Examples in Nature

#### 4.2.1. Antireflective Surfaces

**Figure 10:**
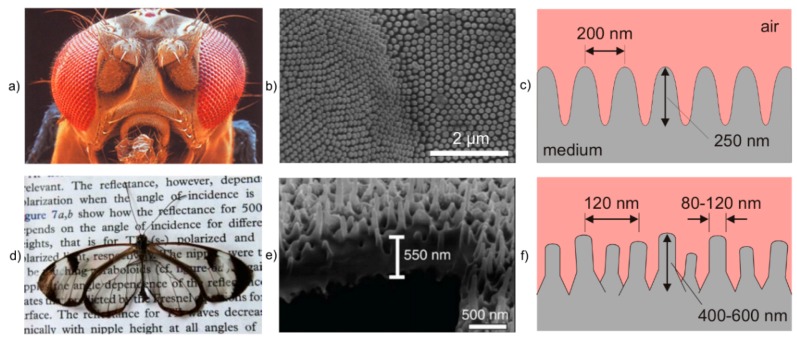
Examples of antireflective surfaces in nature: (**a**–**c**) moth eye and (**d**–**f**) glass wing butterfly *Greta oto*. (**b**) Adapted from Gonzalez and Gordon [77], Copyright 2014, with permission from OSA Publishing; (**d**,**e**) Adapted by permission from Macmillan Publishers Ltd.: Nature Communications, Siddique *et al*. [76], Copyright 2015.

The moth eye is the most prominent example for antireflective surfaces in nature. In 1967 Bernhard described the principle by which the reflection from the corneas of night-flying moths is reduced for the purpose of camouflage [73]. A moth eye antireflection surface consists of a very fine array of nipple-like protuberances with hexagonal packing (Figure 10a–c). These behave as a gradation of refractive index, which substantially reduces the reflectance [67]. It was found, that nipples with a parabolic shape, a distance of 200 nm, and a height of 250 nm, touching each other at the base, completely prevent the reflectance for normally incident light [74]. Thus, the eye glare of moths that are inactive during the day is significantly reduced, and consequently they are less visible for predators. Beyond, the corneal nipples act as an optically matched layer between the facets of the compound eye of the moth and the surrounding air, and maximize the incoupling of light in the eye [68]. Another concept of camouflage motivated antireflective surfaces is followed by the glasswing butterfly (*Greta oto*). This butterfly has transparent wings with very low reflectance over the whole visible spectral range even for large view angles [75,76]. The transparency of the wings makes it difficult for predatory birds to track the butterfly during the flight. The omnidirectional antireflection behavior of the wings is caused by small nanopillars of high aspect ratio that are irregularly arranged and feature a random height and width distribution (Figure 10d–f) [76].

#### 4.2.2. Structural Colors

**Figure 11:**
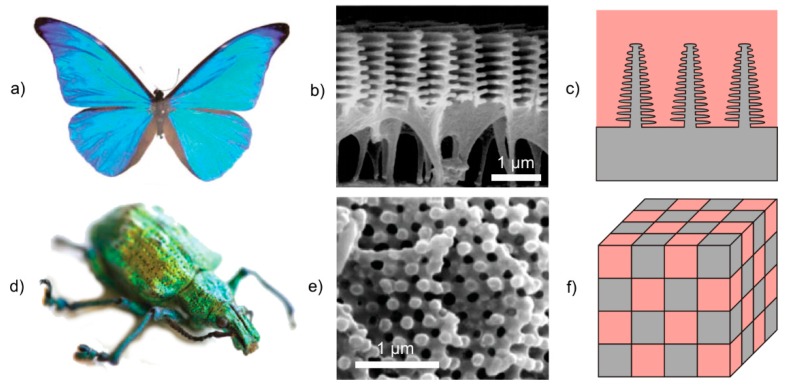
Examples of structural colors in nature: (**a**–**c**) *Morpho* butterfly and (**d**–**f**) *Lamprocyphus augustus* weevil. (**a**) Watanabe *et al*. [83], Copyright 2005 The Japan Society of Applied Physics; (**b**) Adapted with permission from Kinoshita *et al*. [84]; (**d**,**e**) Adapted with permission from Galusha *et al*. [85], Copyright 2010 John Wiley and Sons.

Nature’s colors have three main sources: pigments, structural colors and bioluminescence [69]. While structural colors are the result of selective light reflection, diffraction, colors from pigments and bioluminescence originate from selective light absorption and chemical reaction, respectively [78]. Many types of butterflies use light-interacting structures on their wing scales to produce color for camouflage, thermoregulation, and signaling [79]. Among them, *Morpho* butterflies are the most well-known (Figure 11a–c). The intensely bright iridescent blue coloring in male butterflies facilitates very long-range signaling of up to 500 meters [80]. The structure of the wing scales is characterized by a series of longitudinal ridges that are spanned by cross ribs, which are located on a basal lamellae that is supported by columnar trabeculae [81]. These structures selectively cancel out certain colors through wavelength interference while reflecting others, depending on the exact structure and interspatial distance between diffracting layers [82]. Here, the resulting structural color results from combining different physical mechanisms. A multi-film interference was found in the vertical direction and diffraction grating in the horizontal direction [69].

Examples of natural photonic crystals with a 2D and 3D structure were first found in the sea mouse *Aphroditidae polychaeta* [86] and in the opal weevil *Pachyrhynchus argus* [87], respectively. The Brasilian weevil *Lamprocyphus augustus* is another example of 3D photonic crystals that has attracted much attention due to its iridescent green color with golden reflections (Figure 11d–f). The beetle’s scales consist of cylindrically shaped chitin building blocks and air gaps, which are arranged in the symmetry of a diamond lattice with an average lattice constant of 450 nm [88]. This structure is transparent for all wavelengths except 500 to 550 nm. Thus, it acts as a perfect mirror for green light and makes the beetle appear iridescent [88].

### 4.3. Antireflective LIPSS

**Figure 12:**
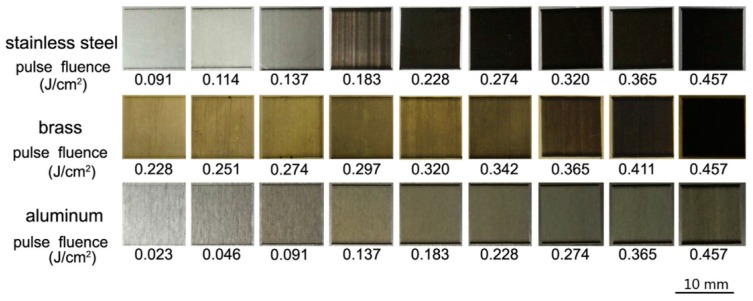
Optical images of metal surfaces (stainless steel, brass and aluminum) treated with fs-laser radiation at different pulse fluences. Reprinted from Ou *et al*. [89], Copyright 2016, with permission from Elsevier.

The elimination of undesired reflections from optical surfaces is a key aspect during the development of many technologies, such as lenses and displays. On the other hand, an enhanced coupling of electromagnetic radiation to a materials surface is of great importance for e.g., solar cells, light sensitive detectors, optoelectronic devices and metal absorbers. Given that a rippled surface is much less reflective and much more absorbing than a smooth surface, LIPSS provide a versatile and relatively simple direct writing approach to enhance e.g. the efficiency of solar cells by boosting their ability to harvest more sunlight. By taking into consideration the broad palette of available laser sources, the relationship between incident laser wavelength *λ* and the resulting structural sizes *Λ* facilitates the realization of LIPSS-based devices suitable for the entire spectral range from the UV up to the near-IR. In this context, the major advantage is given by the feasibility of the grating-like structures on all classes of materials and that the writing method heats only the surface of a material, leaving underlying structures unaffected. Moreover, the optical performance, especially in terms of the period *Λ* and the alignment of the structures, can be adjusted flexibly over a wide range based on the various influencing parameters including the pulse number *N*, the incident angle *θ_i_*, the laser fluence *F* and the polarization state. With increasing *F*, the blackening effect of stainless steel, brass and aluminum surfaces treated with fs-laser radiation increases (Figure 12) [89]. Based on SEM micrographs, Ou *et al*. demonstrated that the blackening threshold of stainless steel and brass is correlated to the appearance of LIPSS. With increasing *F*, the shape of the grating structure is more pronounced and the modulation depth increases. Consequently, the absorbed energy increases due to grating-assisted coupling [22,90] and finally the initially high-reflective surfaces appear completely black to the naked eye.

In dependence on the utilized parameters and the resulting surface morphology, the reflection of such grating-like structures can be reduced down to about 5% for metals [89,91]. In the case of silicon, the most significant decrease in the total reflectance is above the band gap at the wavelength range between 250 and 800 nm, where reflectance is reduced by a factor of about 3.5 in the visible wavelengths and by a factor of about 7 in the UV [92]. Below the band gap, the total reflectance is reduced by a factor of 1.7 compared to an untreated surface. Through the proper scanning of a line-shaped fs-laser beam, Hong *et al*. demonstrated the extension of the area of a uniformly distributed periodic LIPSS-pattern on a silicon substrate [93]. Straightforward and inexpensive, they used a cylindrical lens to widen a fs-laser beam to a width of 50 µm. The resulting homogenous pattern is characterized by a ripple-depth of 300 nm, which is much deeper than the one previously reported. As a result, an absorption enhancement of 41% compared to a planar silicon wafer was achieved. Concerning the realization of photovoltaic devices, the light reflection of silicon including scattering has been further reduced to less than 3% for the entire solar spectrum [94].

However, the morphology of the underlying structures differs remarkably from LIPSS. As described by Nayak *et al*., they are conical shaped with 5–7 µm in base diameter, 8–10 µm in height tapering down to about 100 nm at the tip and separated by about 6 µm from each other. Compared to LIPSS, the fabrication requires a much higher laser fluence and the utilization of reactive background gasses [95].

**Figure 13:**
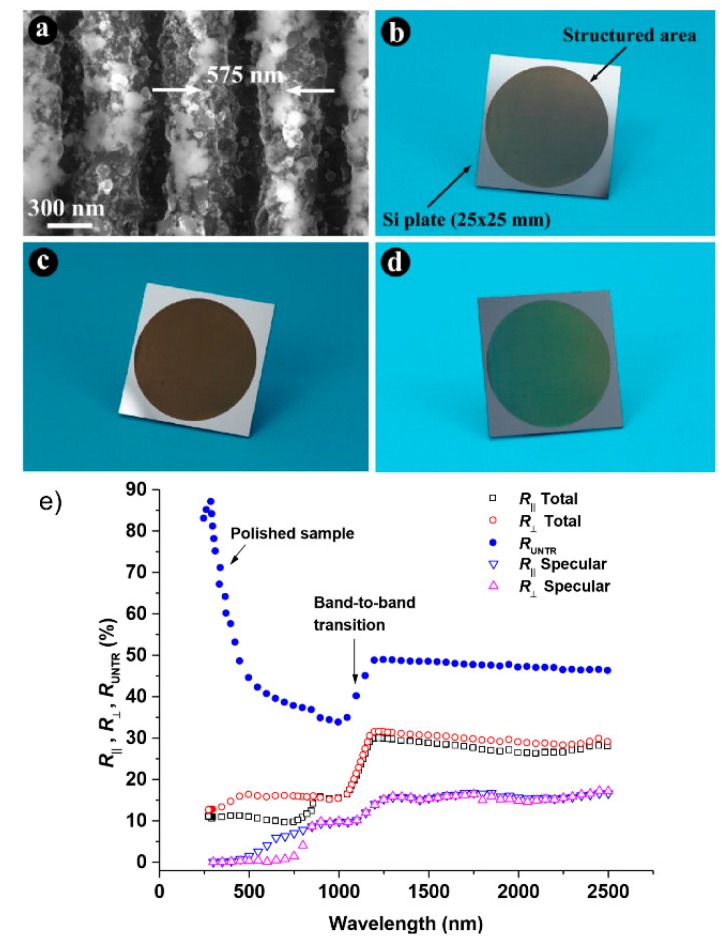
Antireflection effect of LIPSS on silicon: (**a**) SEM micrograph of LIPSS; (**b**–**d**) photographs of laser structured silicon surfaces reveal various shades of dark colors at different viewing angles; (**e**) spectral and polarization response at an incidence angle of 6° of the structured silicon sample. Adapted from Vorobyev and Guo [92], Copyright 2011, with permission from OSA Publishing.

As one of the main characteristics of the grating-like surface structures, polarized reflection measurements with an integrating sphere revealed that the reduction of light reflection is most significant for the incident polarization parallel to the LIPSS grating vector (*i.e.*, the electrical field vector is perpendicular to the grooves) for both total and specular reflectance (Figure 13e) [18,92]. Consequently, the optical response of LIPSS-based surfaces shows an anisotropic behavior concerning the direction and the polarization of incident radiation (Figure 13b–d). For both types of materials (metals and silicon) structured with a grating period of about 600 nm, this effect is most significant in the VIS. Nevertheless, for both polarization directions the reduced reflection can be measured in the entire spectral range. This indicates an influence of the nanoparticles. They are distributed over the grating ridges and increase the fundamental light absorption caused by the subwavelength grating effect (Figure 13a) [96].

**Figure 14:**
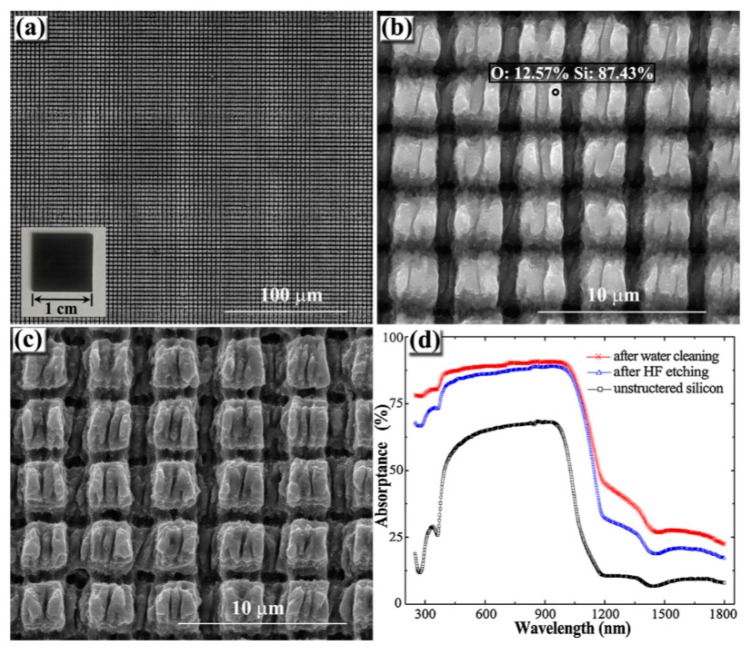
Fabrication of 2D periodic structures on silicon after scanning with fs-laser multi-beams: (**a**) overview of the grid-like structures; (**b**,**c**) enlarged view of the laser-induced surface morphologies before and after HF-etching for 60 min and (**d**) spectral absorptance of the samples. Reprinted from Pan *et al*. [99], Copyright 2016, with permission from Elsevier.

Concerning the further improve of omnidirectional, broadband and polarization-independent optical surfaces, higher-dimensional hierarchical LIPSS-based structures imply great potential and are therefore in the focus of current research. In this context, an obvious idea is related to the fabrication of e.g., crossed grating structures by a multiple scanning procedure with different polarization directions. However, as pointed out by Yao *et al*., a twofold scanning with the linear polarization perpendicular to each other leads to an almost complete replacement of the initial LIPSS pattern, *i.e.*, that the orientation of the grating-like structure is always dictated by the most recent polarization direction [97]. Cong *et al*. demonstrated a direct method to realize such two-dimensional matrix subwavelength dot structures by focusing two-color cross-polarized fs-laser pulses (in this case *λ*_1_ = 800 nm and *λ*_2_ = 400 nm) through an optical lens [98]. The morphology was tuned in a wide range in dependence of the time delay between the corresponding ultrafast dynamic processes. An alternative approach was adopted by Pan *et al*., who fabricated two-dimensional periodic structures on silicon surfaces by fs-laser radiation with the assistance of a microlens array placed in the beam path [99]. By scanning the laser beam twice successively along the *x*- and the *y*-directions at a laser power of 30 mW and a scanning velocity of 150 µm/s in air, grid-like structures were realized (Figure 14a). As illustrated, LIPSS appeared both on the top of the surface as well as in the valleys formed between grooves (Figure 14b,c). The optical absorptance of these “hybrid” structures measured between 250 nm and 1800 nm wavelength indicated an enhancement most significantly below 400 nm and absorption values between 86% and 91% in the visible region (Figure 14d). Despite the promising influence of the beam polarization on the orientation of the grating structures, up to now only a few studies considered the utilization of transient polarization states during LIPSS-based surface structuring. By means of spatial light modulators (SLM) and additional waveplates, Jin *et al*. utilized the dynamical switching between four specific polarization states (linear horizontal and vertical, radial and azimuthal) to demonstrate that a highly controlled real-time nanostructuring of polished stainless steel samples is possible [100]. Nevertheless, the switching was conducted discrete between the mentioned polarization states, whereas the corresponding areas show highly periodic LIPSS that are aligned perpendicular to the electrical field vector. 

Some authors reported about direct fs-laser LIPSS-based surface structuring by using tailored optical vortex beams with different spatial distributions of the polarization state which are generated by means of q-plates [101], SLM [102] and SLM in combination with s-waveplates [103], respectively. It was shown, that the vortex beams facilitate to extend the fabrication of even more complex and unconventional surface structures. Beyond, Gräf and Müller showed for stainless steel, that the well-known relationship between laser beam polarization and the alignment of the emerging structures can be used to continuously control the orientation of the surface textures by the utilization of a continuously rotating electrical field vector [104]. In dependence on its rotation frequency, the originally well-ordered ripple pattern obtained from static linear polarization has been transferred into surface morphologies with a well-defined alignment and degree of disorder. As a main result, the induced surface structures are characterized by their different optical response to a side-illumination at grazing incidence.

### 4.4. LIPSS with Structural Color

Structural coloration is promising in providing brilliant colors, adaptive camouflage, label marking, and optical data storage. Therefore, it is of great potential for numerous industrial, commercial and technical applications. In this context, nature offers a great variety of different examples of functional optical surfaces, which all origin from a well-ordered micro- and nanostructured surface. Based on the study of these biological systems, various technologies have been adopted to transfer nature’s functional principles to potential technical applications. Hereby, LIPSS-based direct laser structuring has emerged as a mask-free, flexible, versatile and contactless tool to modify the optical surface properties of different types of materials. This was shown for the very first time by Vorobyev and Guo in 2008, who demonstrated that the colorizing of surfaces facilitates to control the optical properties, in particular the color, of aluminum, gold and platinum [105]. The most characteristic feature of LIPSS-based surfaces is related to the exhibition of different colors at different viewing angles and to the close interrelation of the colorizing effect to the antireflective surface properties [105]. They both origin from the diffraction behavior of the grating-like surface structures, that is modified by the appearance of homogenously distributed nanoparticles [106]. This grating diffraction origin is substantiated by a red shift of the present color that can be observed with increasing inspecting angle [107].

**Figure 15:**
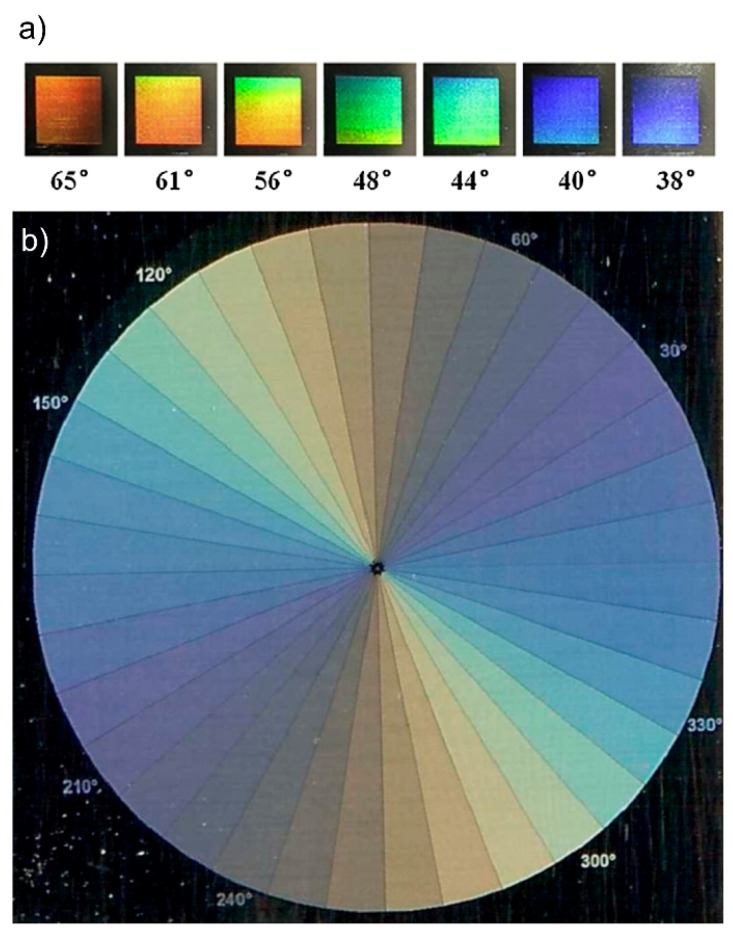
LIPSS-based colorizing of stainless steel: (**a**) variation of structural colors observed at fixed observation angle and different incident angles ranging from 38° to 65° and (**b**) scanned image (1200 dpi) of the sample surface marked by thirty six disc sections characterized by different ripple orientations (from 0° to 350°) but fixed incident and observation angle. (**a**) Reprinted from Yao *et al*. [97], Copyright 2012, with permission from Elsevier; (**b**) Adapted from Dusser *et al*. [113], Copyright 2010, with permission from OSA Publishing.

According to a generally accepted theory, the spatial ripple period is directly related to the initial laser wavelength, *i.e.*, the structural sizes are equal or somewhat smaller than *λ*. The Ti:sapphire laser with a central wavelength of 800 nm is currently the most common type of fs-laser used for LIPSS fabrication. The corresponding ripple period (LSFL) ranges from about 400 to 800 nm. From the physical point of view, the colorizing effect can be controlled by the angle of incidence and observation, the orientation of the ripple pattern relative to the plane of incidence and by the ripple period *Λ* (Equation (7)). This is illustrated in Figure 15 for LIPSS fabricated on stainless steel. It becomes evident for a fixed observation angle, that different colors appear for different angles of incidence (Figure 15a). On the other hand, Figure 15b shows thirty-six disc sections with grating-like structures with a period *Λ* = 660 nm and a height of 200 nm, which are characterized by different ripple orientations reaching from 0° to 350°. As a result, each section exhibits different colors from the visible spectral range for fixed angles of incidence and observation. From the technological point of view, versatile influencing parameters are available to modify the ripple structures and their optical response. By means of the processing parameters including e.g., *F*, *N* and the surrounding atmosphere, the colorizing effect can be finely tuned via the well-defined modification of the induced ripple period. It was shown by several authors for different materials, that *Λ* slightly decreases with increasing *N* hitting a certain surface area [2,22,108,109,110]. In this context, Huang *et al*. demonstrated that the *Λ* -decreasing phenomenon in dependence on *N* is far more prominent in the case of dielectrics and semiconductors than in the case of conductors [22]. Beyond, a moderate increase of *Λ* was observed with increasing *F* [109,110,111]. In the case of silicon, Yang *et al*. demonstrated different spatial periods in dependence on vacuum, nitrogen and air as atmospheres during structuring [112]. Moreover, the colorization effect was most obvious in vacuum, which was attributed to clearer grating structures.

**Figure 16:**
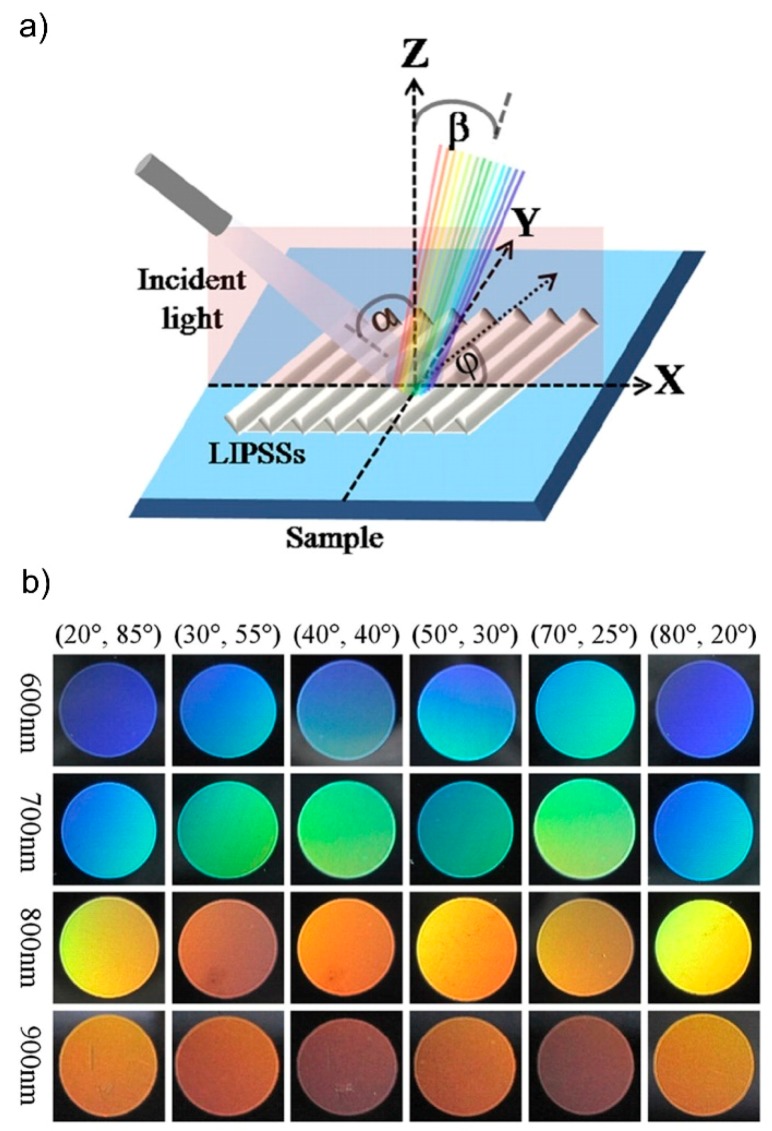
Structural colors formed with different spatial periods: (**a**) principle of the color measuring setup and (**b**) photographs taken at fixed observation angle *β* = 20° (left axis: spatial periods, upper axis: value pair (*α*, *φ*) consisting of *α*—orientation of the incident light to the *Z*-direction and *φ*—ripple direction to *X*-direction). Adapted from Li *et al*. [114], with permission of Springer.

Of course, the variation of the utilized fs-laser wavelength *λ* itself has a much greater impact on the LIPSS period and therefore introduces a greater flexibility to laser color marking. Despite this obvious idea and its promising advantages, color marking with an adjustable laser wavelength is only less investigated so far. Li *et al*. [114] systematically studied LIPSS fabrication utilizing different laser wavelengths between 400 nm and 2200 nm by means of an optical parametric amplifier. As illustrated in Figure 16b for stainless steel, the investigated approach facilitates to considerably expand the diversity of available colors. In addition to the fact different that for a given ripple period different colors appear at different coordinates (*α*, *φ*) (see Figure 16a), the reported enhancement of the spectral range is supported by different colors appearing at the same coordinates for different ripple periods.

The strong dependence of the ripple orientation on the polarization of laser light offers the opportunity of decorating different regions of the surface with different types of ripples. As shown by Yao *et al*., different patterns can be selectively displayed with structural color when white light is irradiated on the surface from different directions [97]. Moreover, they demonstrated the possibility of decorating the same region with two or more types of ripples with different orientation, which facilitates to selectively display different patterns with spatial overlapping with structural color. Possible applications are related e.g., to decoration encryption. Finally, the continuous change of the orientation of the ripple pattern as demonstrated by Gräf and Müller by means of a well-defined control of the electrical field vector during surface scanning might enable to realize a color gradient at fixed angles of incidence and observation [104].

## 5. Drag and Friction

### 5.1. Theoretical Aspects

Any object that moves relative to the flow of a gas, a liquid or is in contact to another solid surface in motion, faces a force that acts opposite to its relative motion. In the case of a fluid, this force is called drag force *F_D_* and can be calculated according to:

(9) $$F_{D}=\frac{\rho \times v^{2}}{2}\times c_{D}\times A$$

where *ρ* is the density of the fluid, *v* is the velocity of the object relative to the fluid, *A* is the cross sectional area of the object, and *c_D_* is the drag coefficient. *c_D_* depends on the shape of the object and on the Reynolds number Re_x_:

(10) Rex=v×x×ν−1

where *x* represents a linear dimension in the direction of the flow and *υ* is the kinematic viscosity of the fluid. *c_D_* amounts to 1.328 (Re_x_)^−0.5^ and 0.455 (logRe_x_)^−2.58^ for laminar and turbulent flow, respectively [115]. It was shown, that the viscous drag of turbulent boundary layers is the main source of high *F_D_*, and that riblets are a versatile tool to reduce viscous drag [116]. Experiments in an oil channel with adjustable blade riblets have shown that the optimal riblet height *h* is at 0.5 s, with *s* representing the distance between adjacent riblets [117]. With this surface a turbulent shear stress reduction of 9.9% below that of a smooth surface has been achieved [117].

If two moving solid surfaces come in contact to each other, dry friction resists their relative lateral motion. This may lead to a degradation or damage of the related materials by wear. The force of friction *F_F_* can be calculated by:

(11) $$F_{F}\leq \mu \times F_{N}$$

where *F_N_* is the normal force that compresses two faces together and *µ* is the coefficient of friction. As the crucial parameter, *µ* is influenced by a great variety of different parameters such as the surface roughness, possible lubricants, the surface chemistry, the contact area and stress, the sliding speed as well as environmental conditions.

### 5.2. Examples in Nature

**Figure 17:**
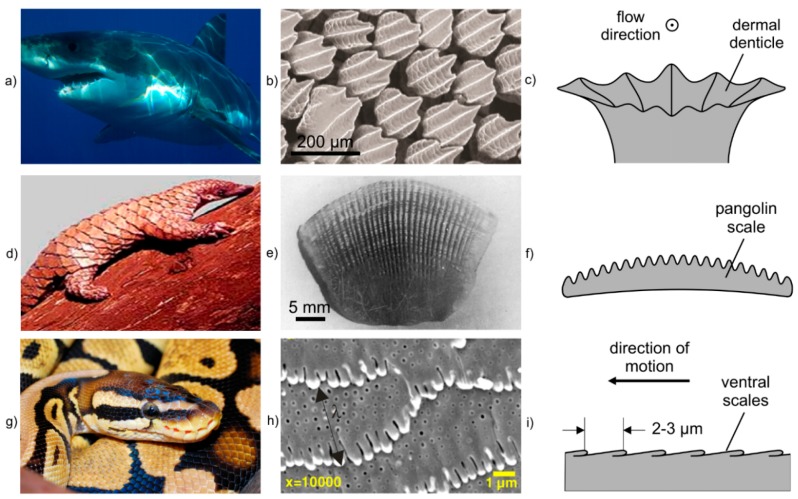
Examples of tribological surfaces in nature: (**a**–**c**) skin of sharks; (**d**–**f**) pangolin *Manis tricuspis*, and (**g**–**i**) *Python regius*; (**b**) Adapted with permission from Wen *et al*. [127]; (**d**) Adapted from Ofusori *et al*. [128]; (**e**) Adapted from Tong *et al*. [129] with permission from Springer; (**h**) Reproduced with permission from Abdel-Aal and El Mansori [130] Copyright IOP Publishing. All rights reserved.

In 1936 the British zoologist James Gray hypothesized that the skin of dolphins must have special drag reducing properties, that facilitate to swim at much higher speed and acceleration than the physiological condition of the dolphin should allow (“Gray´s paradox”) [118]. Since then many examples of drag reducing marine organisms e.g., barracuda [119], tuna [120], boxfish [121], penguin [122], and shark [123] were investigated and the related drag reducing mechanisms were explained. It was shown, that the skin of different species of fast sharks is structured in a very similar manner (Figure 17a–c). It consists of tiny scales with parallel riblets that are aligned in the direction of flow [124,125]. The size of the scales is in the range from 100 to 200 µm [126]. The riblets have a height of 20 to 30 µm [126], and the number of riblets per scale varies from 1 to 7 for different species [124].

Soil-burrowing animals like dung beetles, ground beetles, and the pangolin dig holes in the ground to live in [131]. Therefore, their skin must be effectively resistant against friction and abrasive wear. For this purpose, the body of the pangolin is covered with a layer of keratin scales, each about 25 mm × 40 mm in size (Figure 17d–f) [132]. These scales are structured by riblets that are aligned in the longitudinal direction of the pangolin. The riblets have a height around 400 µm and a distance to each other in the range of 400–800 µm. It was shown, that the free abrasive wear of the scale surface is a function of the orientation of the riblets with respect to the sliding direction of the abrasive particles, the abrasive particle size and the relative sliding velocity [133]. Depending on their size, particles can either be guided along the direction of the riblets (“Guiding-Effect”), or can roll between them (“Rolling-Effect”) [133].

In the course of evolution squamate reptiles like snakes developed customized responses to a broad variety of tribological conditions that allows them to survive even in the remotest corners of the earth. The skin of snakes exhibits hierarchical submicron and nanoscale features that facilitate the reduction of adhesion and exhibit abrasion resistance as well as frictional anisotropy which are fundamental requirements for their movement [130]. Particularly the frictional anisotropy must be kept over a long period of time until new skin is molted. This challenging problem is intensified by the continuous contact of the ventral body of the snake with the ground. In addition to the surface topography, the frictional properties depend on the surface energy and the materials properties. Regarding the mechanical properties, Klein and Gorb [134] revealed that the layered structure of the skin consists of a hard, robust and inflexible outer surface and softer and flexible inner layers. From the topological point of view, the ventral side of the reptile comprises hexagonal scales that are elongated along the lateral axis of the reptile and contain micro fibril structures (Figure 17g–i). Their ordered directional texture and the shape induce high friction anisotropy for enhancing forward motion with minimum adhesive forces and friction [135].

### 5.3. Tribological LIPSS

In recent decades, surface engineering in terms of the tribological properties has become an important tool to reduce wastage of resources resulting from high friction and wear, to decrease CO_2_ emission and to improve wear resistance of technical surfaces. Potential applications are related to the fields of e.g., aerospace, automotive, energy, ships, textiles and mechanical engineering [126]. The surface roughness in particular determines the contact area and stress as well as lubricant paths and reservoirs. In this context, it was shown by several authors that hierarchical surfaces produced by laser interference patterning with feature sizes in µm-range can significantly tailor the tribological properties of surfaces [136,137,138]. As first proposed in 1999 by Yu and Lu [139], the introduction of LIPSS-based micro-and nanostructures also provides a promising approach to improve the tribological performance of surfaces. Some years later, Mizuno *et al*. demonstrated the reduction of *µ* based on LIPSS structuring of diamond-like carbon (DLC) [140]. For this purpose, an atomic force microscope with a spherical glass tip was used under well-defined atmospheric conditions and low normal loads in the range of nN to measure *µ* in different moving directions relative to the LIPSS pattern. The reduction of *µ* was attributed to a decrease of the contact radius and to an increase of the distance between the DLC coated surface and the spherical glass tip. Consequently, the interacting area decreases and leads to remarkably lower adhesions forces [140].

**Figure 18:**
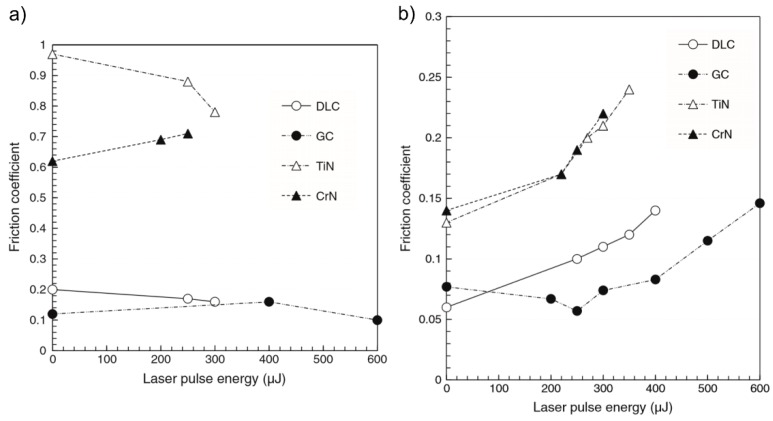
Friction coefficients of DLC, GC, TiN and CrN measured as a function of laser pulse energy using (**a**) a load of *W* = 2 N and a steel ball and (**b**) an ultralight load of *W* = 1.5 mN and a diamond tip. Reprinted from Yasumaru *et al*. [142], Copyright 2011, with permission from Elsevier.

It was shown, that the influence of the testing directions on the friction is negligible compared to the influence of the contact area and the adhesion force. The analysis of structured DLC-films coated with a molybdenum disulfide (MoS_2_) layer using a ball-on-disc friction test machine with different loads (*W* = 2 N and 10 N) and balls (bearing steel and WC-Co with a diameter of 6 mm) revealed an excellent frictional performance [141]. The corresponding reduction of *µ* down to 0.02 was explained by the LIPSS pattern providing lubricant reservoirs. Furthermore, a laser-induced transformation of the DLC-films to glassy carbon (GC) was detected, which positively effects the bonding of the MoS_2_-layer and the DLC-film [141]. The extension of the investigations of Yasumaru *et al*. to nitrides (TiN, CrN) revealed a higher friction of the nitrides when compared to carbon materials, which was explained by the low adhesive nature of the carbon materials (Figure 18) [142]. Contrary to micro friction analysis with a load of 1.5 mN and a diamond tip (Figure 18b), the friction coefficient decreases with increasing laser pulse energy during the macro friction analysis using a load of 2 N and a steel ball (Figure 18b). Pfeiffer *et al*. demonstrated a significant reduction of *µ* and the corresponding wear rate resulting from fs-laser structuring of super-hard tetrahedral amorphous carbon films [143]. The films where tested under non-lubricant conditions by using a pin-on-disk-tribometer. 

In addition to structured surface coatings, Eichstädt *et al*. investigated the influence of LIPSS on the friction behavior of bulk materials [144]. For this purpose, silicon was structured with a ps-laser (*λ* = 1030 nm) resulting in LIPSS with a period *Λ* = 750 nm. The analysis of the surfaces under non-lubricant conditions and normal loads in the range of mN with a ball-on-flat-configuration revealed an increased friction coefficient *µ*. However, this effect could not be explained completely only by geometrical facts, interacting surfaces and altered material properties due to laser structuring. Wang *et al*. [145] investigated the friction properties of large area metal surfaces structured with fs-laser pulses. As a main parameter, they varied the measuring direction of the ball-on-disk tribometer (load of 2 N) relative to the LIPSS orientation. In the case of stainless steel, Wang *et al*. [145] observed a significant reduction of the friction of LIPSS-based surfaces (*Λ* = 550 nm) when compared to smooth surfaces under dry conditions as well as with the ester based lubricant PriolubeTM. This reduction was explained by a combination of a lower adhesion, an asperity deformation, and an accumulation of trapped wear particles in the grooves of the LIPSS pattern.

**Figure 19:**
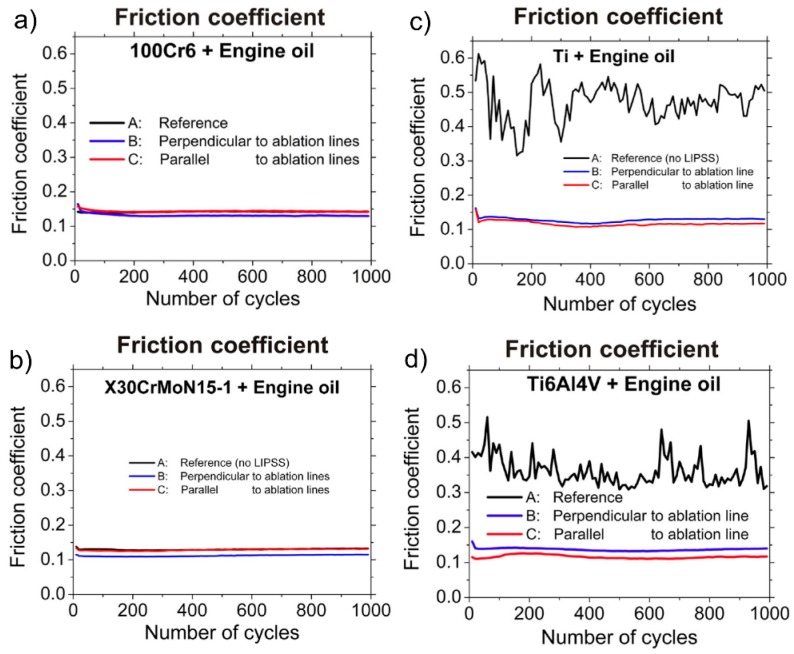
Friction coefficient *µ* as a function of the number of cycles as obtained during reciprocal sliding (normal force 1 N, stroke 1 mm, frequency 1 Hz) of fs-laser structured surfaces in engine oil against a 100Cr6 steel ball: (**a**) 100Cr6; (**b**) X30CrMoN15-1; (**c**) pure titanium; and (**d**) Ti6Al4V. (**a**,**d**) Adapted from Bonse *et al*. [13], with permission of Springer; (**b**,**c**) Reprinted from Bonse *et al*. [146], Copyright 2015, with permission from Elsevier.

Bonse *et al*. investigated the tribological performance of LIPSS on different metal surfaces including commercial pure titanium, titanium alloy Ti6Al4V, high toughness bearing steel X30CrMoN15-1 and stainless steel 100Cr6 (Figure 19) [13,146]. For the processing of large area LIPSS (5 mm × 5 mm) in air, linear polarized fs-laser pulses with a peak fluence of *F* = 0.11 J/cm^2^ and a pulse duration of 30 fs were used. It was shown, that reciprocal sliding against a ball of hardened 100Cr6 steel (diameter: 10 mm) utilizing paraffin oil as lubricant for 1000 sliding cycles at 1 Hz with a normal load of 1 N had no beneficial influence on all investigated metals. In contrast, a significant reduction of wear and the friction coefficient were observed for sliding of pure titanium (Figure 19c) and Ti6Al4V (Figure 19d) in engine oil. This effect was attributed to the presence of additives efficiently covering the laser structured surfaces. However, it is less pronounced for the investigated steel surfaces (Figure 19a,b) when compared to the titanium metals. 

**Figure 20:**
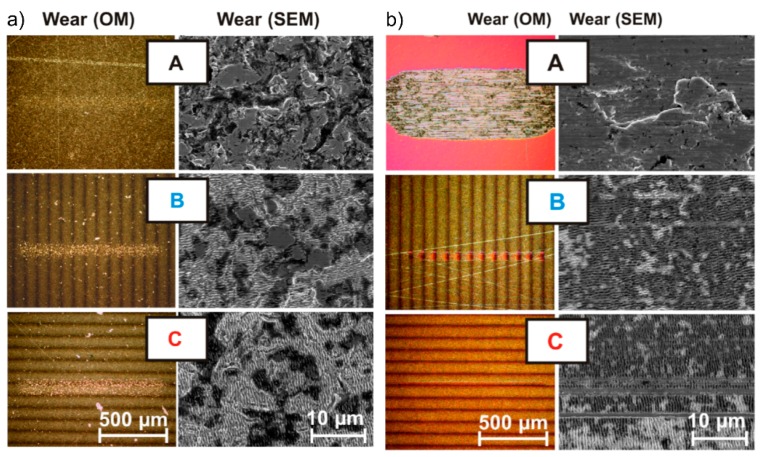
Optical micrographs (OM) and SEM micrographs (SEM) of the corresponding wear tracks taken after tribological analysis using engine oil: (**a**) X30CrMoN15-1; (**b**) pure titanium; (**A**) reference (no LIPSS); (**B**) perpendicular to ablation lines; (**C**) parallel to ablation lines. Reprinted from Bonse *et al*. [146], Copyright 2015, with permission from Elsevier.

This tendency is strengthened by wear tracks of X30CrMoN15-1 and pure titanium taken after tribological analysis perpendicular (B) and parallel (C) to the ablation direction (Figure 20). In the case of X30CrMoN15-1, SEM micrographs reveal a small damage in the wear tracks (Figure 20a). In contrast, the wear tracks on pure titanium are very small when compared to a reference (A) (Figure 20b). Moreover, the corresponding SEM micrographs reveal that the LIPSS pattern is still existent, even if there are single scratches arising from tribologically abraded particles [146].

Based on the previous studies concerning LSFL, Bonse *et al*. [147] extended their investigations to the influence of large area structured surfaces containing HSFL on titanium. The corresponding LIPSS pattern with a significant smaller period of 80 nm and a depth of 30 nm where fabricated with a wavelength *λ* = 790 nm, a pulse duration of 30 fs and a peak fluence *F* = 0.08 J/cm^2^. The reciprocal sliding tribological analysis performed analog to the LSFL with paraffin and engine oil as lubricants revealed, that HSFL have no effect on the friction behavior. From the destroyed surface after tribological analysis, the authors conclude that the depth of the LIPSS plays a very important role and that a positive friction behavior requires surface structures deeper than the sample deformation induced by the tribological tests.

**Figure 21:**
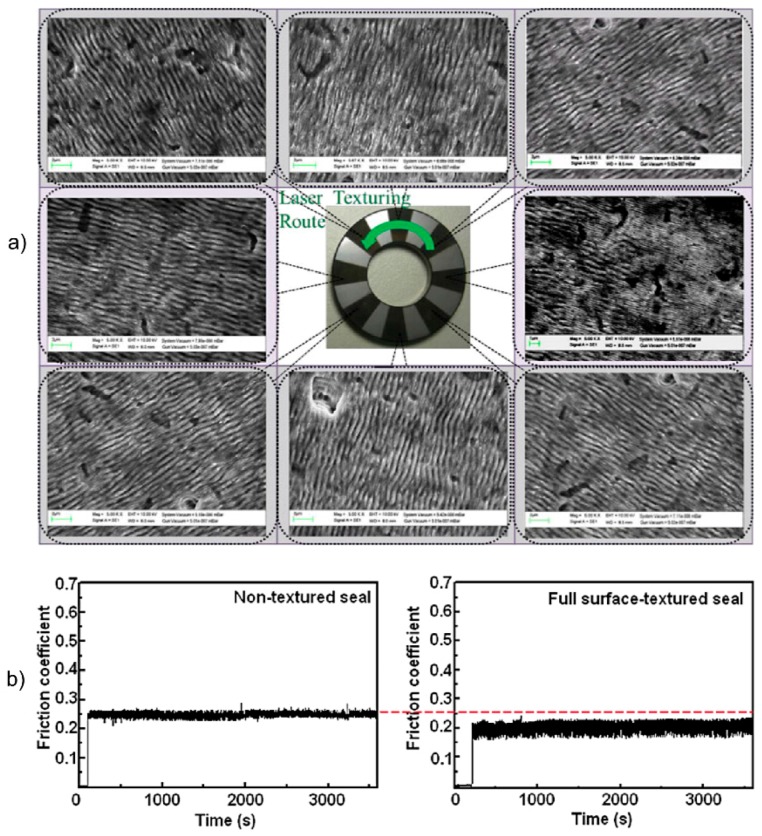
Friction properties of SiC mechanical seals: (**a**) SEM micrographs of the surface and (**b**) friction coefficient of the unstructured (**left**) and full-textured seal (**right**). Adapted from Chen *et al*. [148], with permission of Springer.

A specific application was studied by Chen *et al*. [148], who structured silicon carbide (SiC) mechanical seals with microscale strips and periodic nanostructures (Figure 21a). Using a ring-on-disk tribometer with a load of 50 N under atmospheric conditions and deionized water as lubricant, the friction coefficient of structured seals was reduced by 20% when compared to unstructured seals (Figure 21b). This reduction was attributed to a hydrodynamic load of the rotating seal, which occurs due to an infiltration of the nanostructures with water. As a main effect, the contact angle *θ* between the SiC seal and water increases, which on the one hand improves the tribo-chemical reaction of SiC rotating in water and the other hand positively affects the friction behavior [149].

## 6. Future Perspectives

LIPSS proved their suitability to mimic specific features of biological surfaces. By varying materials, laser parameters, and post-treatments it is possible to successfully generate superhydrophobic, anti-reflective, colored, and drag resistant surfaces, respectively. These are of particular interest for numerous industrial, commercial and military applications including e.g., self-cleaning surfaces, fog harvesting, adaptive camouflage, efficient optical switches, drag reducing surfaces, antifouling, and cutting tools to name only a few.

Anti-reflective surfaces could be further adapted to improve the light collection in solar cells or to enhance the performance of optical, optoelectronic and electro-optical devices, such as glasses, mirrors, lenses, photodetectors, surface-emitting lasers, displays, and optical sensors [76]. In the context of light emitting diodes (LED) it was shown, that the light output power of LIPSS-LED was increased by 30% when compared to conventional LED’s at an injection current of 20 mA. This enhancement of the light output power was attributed to the higher amount of photons escaping from the increased surface area of textured GaN-based LED [150]. An actual challenge that could be overcome by utilizing tailored-disordered LIPSS is to engineer broadband plasmonic surface structures that depend only weakly on frequency and polarization and that are characterized by a high angular tolerance of the absorption [151].

The adjustable wetting behavior and alignment of LIPSS might be of particular interest for biomedical applications to control cell adhesion, proliferation and differentiation [152]. Recently, it was shown, that LIPSS enhance the bioactivity of materials in simulated body fluid by reducing the contact angle [153], and by creating functional surface sites capable for *in vitro* apatite formation [154]. Beyond, LIPSS might be a promising alternative to create antibacterial surfaces [155]. Superamphiphobic surfaces might be of particular interest for applications in microfluidics [156], for the synthesis of polymeric particles [157,158], and for the oxygenation of blood [159].

In the future, it seems promising to combine several of the before mentioned properties to generate multifunctional surfaces that combine outstanding optical, tribological and wetting properties, e.g., butterfly wings do not only have beautiful colors, they are also superhydrophobic; shark skin does not only reduce drag, it also represents an effective antifouling surface. Beyond, it might be promising to extend the existing knowledge in LIPSS generation to yet unexplored materials and material combinations like elastomers and multilayer structures to generate e.g., new gecko-inspired adhesive tapes or chameleon-like surfaces capable of changing color.
